# Basrah Preliminary Experience With COVID-19: A Report on 6404 Patients

**DOI:** 10.7759/cureus.13012

**Published:** 2021-01-30

**Authors:** Saad Hammadi, Abbas K AlKanan, Mohammad Fares, Nihad K Mohammed, Ali Raheem Hashim, Awatif Habeeb, Abbas A Mansour

**Affiliations:** 1 Internal Medicine, College of Medicine, University of Basrah, Basrah, IRQ; 2 Radiology, Basrah Health Directorate, Basrah, IRQ; 3 College of Engineering, University of Basrah, Basrah, IRQ; 4 Preventive Medicine, Community Medicine, Basrah Health Directorate, Basrah, IRQ; 5 Medicine, Basrah Teaching Hospital, College of Medicine, University of Basrah, Basrah, IRQ; 6 Epidemiology and Public Health, College of Science, University of Basrah, Basrah, IRQ; 7 Diabetes and Endocrinology, College of Medicine, University of Basrah, Basrah, IRQ

**Keywords:** covid-19, case fatality rate, basrah, iraq

## Abstract

Background: The first case of coronavirus disease 2019 (COVID-19) reported in Basrah was in early March 2020. This study aimed at assessing some of the characteristics of patients with COVID-19 in Basrah during the period from March 4th to September 8th, 2020.

Methods: Retrospective analysis of the University of Basrah database on COVID-19. All patients with positive COVID-19 reverse transcription polymerase chain reaction (RT-PCR) test during the study period were enrolled.

Results: Of 6404 patients included (males 54.8% and females 45.2%), healthcare workers constituted 11.4%. Physicians represented 16.1% of health care workers. The mean age was 39±16.7 years, those aged 61 years or more constituted 9.8%. The case fatality rate was 3% (males 55.2% and females 44.8%). No deaths were reported in adolescents or children. The highest death rate was among those aged 61 years or more.

Conclusion: The situation of COVID-19 infection in Basrah, Iraq is evolving similar to other countries. Studies are needed to assess the influence of associated comorbidities, results of treatment regimens used and variables associated with high mortality.

## Introduction

On March 11, 2020, the World Health Organization (WHO) declared coronavirus disease 2019 (COVID-19) as a pandemic after its first appearance in Wuhan, China, in December 2019. This pandemic uncovered major threats to the health systems of all countries. Clearly, it shows how fragile the health structures are worldwide. The high number of healthcare workers infected till now despite all the recommendation of personal protections uncovers disastrous gaps in the knowledge on the disease. The care for patients with noncommunicable diseases faced a tragic situation because of the shift of almost all care toward patients with COVID-19 [1].

The social distancing caused an unpreceded direct and indirect psychological trauma including the direct negative effect on the economy worldwide [2]. A paucity of symptoms at the early stages of COVID-19, wide range of the incubation period (2-14 days), and false negative reverse transcriptase PCR (RT-PCR) tests were the major obstacles to contain the outbreak by imposing quarantine [3]. The city of Basrah, southern Iraq, reported the first case of COVID-19 on March 9, 2020, and the first death was reported on March 10 [4].

By the end of February 2020, the health authority of Basrah (Basrah Health Directorate) in collaboration with the University of Basrah started to modify the local health system in the city to accommodate for this new disease including preparing one major hospital (Basrah Teaching Hospital) to receive COVID-19 cases, thereafter more than five hospitals became equipped to deal with patients infected with COVID-19.

By mid-June, it was decided to resume care for non-COVID-19 patients because the number of cases from February to mid-June was not very high (around 10-20 cases daily). Unfortunately, this coincided with a sudden increase in the number of newly discovered cases, up to 250-350 daily. Thereafter, there were periods of alternating lockdown and relaxation until September 24, when the lockdown was totally lifted.

In the period from February to mid-June 2020, almost all COVID-19 cases were obliged by the health authority to be hospitalized for at least 10 days. Each patient needed to be twice RT-PCR negative before discharge. After that period quarantine at home became the standard policy because of the predominance of mild cases.

Noteworthy, diabetes mellitus, especially that requiring insulin use, is considered a strong risk factor for progression of COVID-19. Iraq is one of highest prevalence countries for diabetes (reaching up to 20% in Basrah). This could explain the increased number of severe cases in Basrah [5].

This study aimed to assess some characteristics of patients with COVID-19 in Basrah for the period from March up to September, 2020.

## Materials and methods

Design

Retrospective analysis of the University of Basrah database was done for the period between March and September 2020. The study was approved by the ethical committee of the University of Basrah (approval number 56/35/22).

Participants

All RT-PCR positive patients during this period were enrolled. Data collected by the University of Basrah.

The following patients were excluded from the analysis: patients having negative chest computerized tomography (CT) and RT-PCR test, those who died at home with no known RT-PCR status, and those treated in the private sector clinics based on only inflammatory markers and/or CT scan of the chest with no documented RT-PCR status. Variables assessed were age, gender, occupation, and residency.

Laboratory analysis and imaging

In the period from March to the end of April, the samples were sent to the Ministry of Health in Baghdad to perform the RT-PCR. Thereafter, many local laboratories were established in Basrah providing RT-PCR results within 48 hours using the WHO standard.

Data analyses

Data are expressed as number and percentage of mean ± SD accordingly.

## Results

The total number of patients included was 6404. Males constituted 54.8%; 33.6% were employed, and 75.3% of women were housewives. There were 11.4% of cohort healthcare workers; 16.1% of them were physicians (Table 1).

**Table 1:** Basic demographic data of 6404 patients with COVID-19 in Basrah

			N (%)
Gender	Males		3489(54.8)
	Females		2915(45.2)
Job	Employed (All)		2154(33.6)
	Non employed		4250(66.4)
	Healthcare workers	ALL	733/6404 (11.4)
		Physicians	118/733(16.1)
		Others	615/733(83.9)
	Housewives		2194/2915 (75.3)**
	Employed Females		721/2915 (24.7)
Residency	City center		2369 (37.0)
	Peripheries		4035 (63)

The mean age was 39±16.7 years (mean age for males 39.9±16.2 years and for females 38.8±17.5) as seen in Table 2. Those younger than 20 years constituted 12.4% while those aged 61 years or more constituted 9.8% only.

**Table 2:** Distributions of 6404 patients with COVID-19 in Basrah according to age group

		N (%) or mean ±SD
Mean age years	Mean ±SD	39±16.7
Mean age males	Mean ±SD	39.9±16.2
Mean age females	Mean ±SD	38.8± 17.5
Age distribution	Less than 20 years	792 (12.4)
	21-30	1336 (20.8)
	31-40	1336 (23.2)
	41-50	1314 (20.5)
	51-60	894 (13.96)
	61 or more	631 (9.8)

About 63% were from peripheral city districts. There was a case fatality rate of 3% with more deaths in males than women (55.2% and 44.8%, respectively). No deaths were reported in adolescents or children. The highest mortality rate was among those aged 61 years or more (45.8%) as seen in Table 3.

**Table 3:** Distributions of death among 6404 patients with COVID-19 in Basrah

		N (%)
Death according to gender	Males	106(55.2)
	Females	86(44.8)
Death according to age group distribution	Mean age for all Mean ±SD	58.4±14.8
	Mean age for males Mean ±SD	57.6±14.7
	Mean age for females Mean ±SD	59.4±14.8
	Less than 20 years	0(0.0)
	21-30	6(3.1)
	31-40	15 (7.82)
	41-50	37 (19.2)
	51-60	44 (22.9)
	61 or more	88 (45.8)
Total deaths	192/6404(3)	

## Discussion

Underdoing RT-PCR testing is a problem all over the world and only one-tenth of symptomatic patients with serology positive reported previous nasopharyngeal swabs done [3,6]. Furthermore, 40% of COVID-19 cases may be asymptomatic, making the true incidence and prevalence almost impossible to be exactly determined by any study as is the mortality rate [7]. Published data on COVID-19 in Iraq is very limited [8-12].

We report here that males were slightly more affected than females, unlike reports from Saudi Arabia where males contributed to 80% of cases [13,14]. Healthcare workers represented around one-tenth of the total infected patients. Worldwide, among 2,035,395 individuals, there were 99,795 healthcare workers infected (adjusted HR 11·61, 95% CI 10·93-12·33) [15].

One-tenth of cases were adolescent and children and the peak age of affected patients was 31-40 years. In Saudi Arabia 90% of cases were adults and only 10% were children or elderly, while in the western countries, the peak age is 20-29 years [14,16].

There were more deaths in males compared to women. Furthermore, around half of deaths were in those aged 61 years or older with a case fatality rate of 3%. The case fatality was very low in Saudi Arabia (around 0.2%) and in other countries ranging 2.7-7% according to the disease waves [17,18].

This study has its own limitations. First, the prevalence of COVID-19 infection was likely underestimated due to multiple factors, including the fact that most people with symptoms compatible with COVID-19 did not undergo RT-PCR testing and that most mild to moderate cases were treated in the private sector without RT-PCR testing. In addition, many deaths at home were not labeled as COVID-19 because no tests were performed. Furthermore, this report did not assess patient comorbidities and smoking status.

## Conclusions

As in other countries, the status of COVID-19 infection in Basrah, Iraq, is evolving. Studies are needed to assess the influence of associated comorbidities, the results of treatment regimens, and the variables associated with high mortality.

## References

[1] Volpe M, Gallo G (2020). COVID-19 and the forgotten majority. High Blood Press Cardiovasc Prev.

[2] Razai MS, Oakeshott P, Kankam H, Galea S, Stokes-Lampard H (2020). Mitigating the psychological effects of social isolation during the COVID-19 pandemic. BMJ.

[3] Coleman JJ, Manavi K, Marson EJ, Botkai AH, Sapey E (2020). COVID-19: to be or not to be; that is the diagnostic question. Postgrad Med J.

[4] (2020). Iraq: COVID-19 Situation Report No.1, 27 February 2020. https://reliefweb.int/report/iraq/iraq-covid-19-situation-report-no1-27-february-2020.

[5] Mansour AA, Al-Maliky AA, Kasem B, Jabar A, Mosbeh KA (2020). Prevalence of diagnosed and undiagnosed diabetes mellitus in adults aged 19 years and older in Basrah, Iraq. Diabetes Metab Syndr Obes.

[6] Pollan M, Perez-Gomez B, Pastor-Barriuso R (2020). Prevalence of SARS-CoV-2 in Spain (ENE-COVID): a nationwide, population-based seroepidemiological study. Lancet.

[7] Brown TS, Walensky RP (2020). Serosurveillance and the COVID-19 epidemic in the US: undetected, uncertain, and out of control. JAMA.

[8] Allawi JA, Abbas HM, Rasheed JI (2020). The first 40-days experience and clinical outcomes in the management of coronavirus covid-19 crisis. Single center preliminary study. J Fac Med Baghdad.

[9] Sarhan AR, Flaih MH, Hussein TA, Hussein KR (2020). Novel coronavirus (COVID-19) outbreak in Iraq: the first wave and future scenario [Preprint]. medRxiv.

[10] Gorial FI, Mashhadani S, Sayaly HM (2020). Effectiveness of ivermectin as add-on therapy in COVID-19 management (pilot trial) [Preprint]. medRxiv.

[11] Habib OS, AlKanan AK, Abed AH, Mohammed NQ (2020). Epidemiological features of COVID-19 epidemic in Basrah Province-Southern Iraq-first report. Med J Basrah Univ.

[12] Habib OS, Jassim HA, Alshihaby WJ, Mohamed MA (2020). The dynamics of COVID-19 epidemic in Basrah-second report. Med J Basrah Univ.

[13] Jin J-M, Bai P, He W (2020). Gender differences in patients with COVID-19: focus on severity and mortality. Front Public Health.

[14] Alyami MH, Naser AY, Orabi MA, Alwafi H, Alyami HS (2020). Epidemiology of COVID-19 in the Kingdom of Saudi Arabia: an ecological study. Front Public Health.

[15] Nguyen LH, Drew DA, Graham MS (2020). Risk of COVID-19 among front-line health-care workers and the general community: a prospective cohort study. Lancet Public Health.

[16] Venkatesan P (2020). The changing demographics of COVID-19. Lancet Respir Med.

[17] Boretti A (2020). COVID-19 fatality rate for Saudi Arabia, updated 3 June 2020. J Glob Antimicrob Resist.

[18] Ghayda RA, Lee KH, Han YJ (2020). Estimation of global case fatality rate of coronavirus disease 2019 (COVID-19) using meta-analyses: comparison between calendar date and days since the outbreak of the first confirmed case. Int J Infect Dis.

